# Supplementary figures and images for: Human T-Lymphotropic virus type 1 and human immunodeficiency virus co-infection in rural Gabon
Source: PLoS One. 2022 Jul 22;17(7):e0271320. doi: 10.1371/journal.pone.0271320 (PMC9307203; doi:10.1371/journal.pone.0271320)

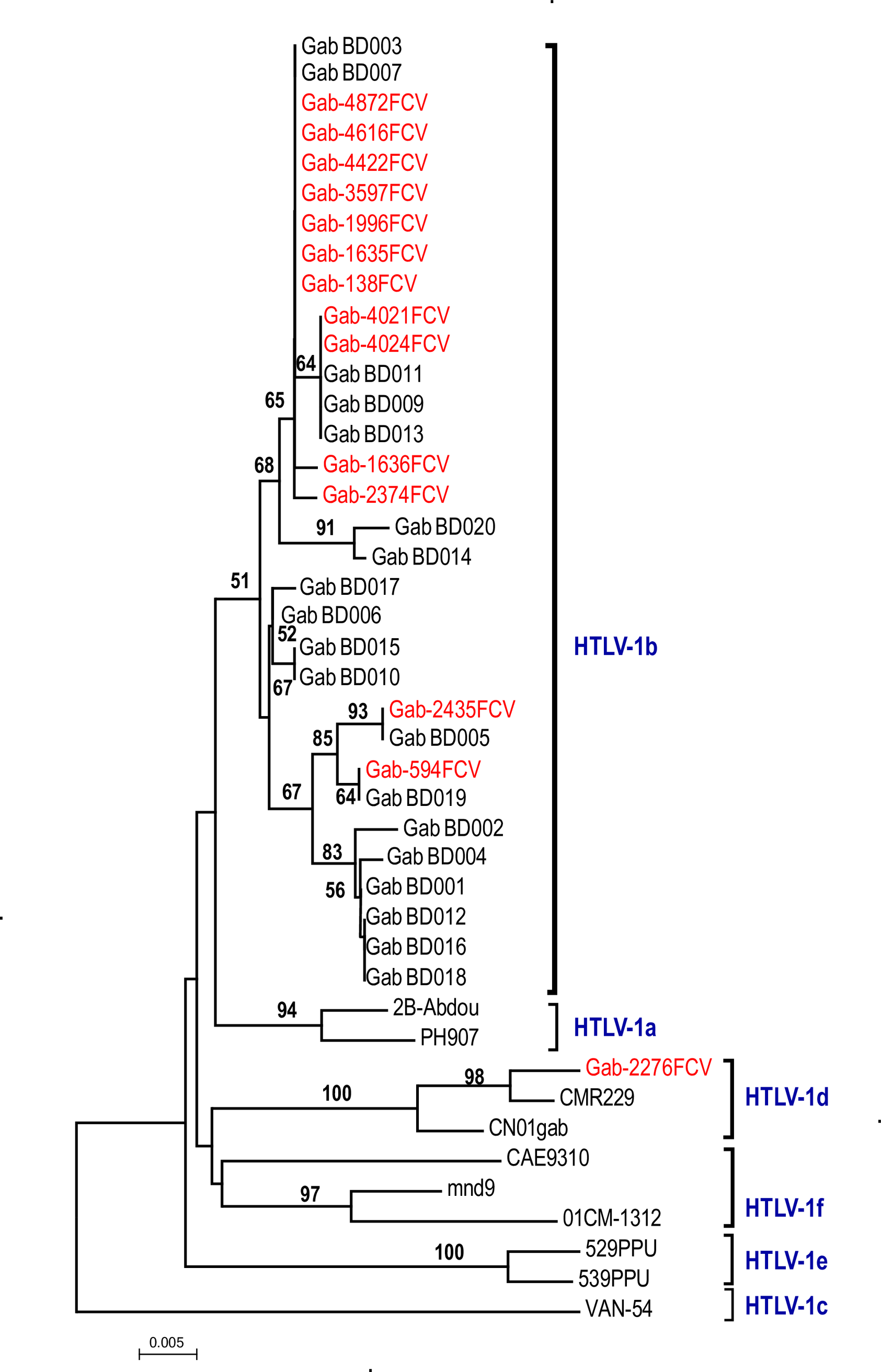

Supplement: S1 Fig — Phylogenetic comparisons were performed with the 522-nucleotide env gp21 gene fragment obtained from 43 HTLV-1 isolates, including the 14 sequences from coinfected HIV-1/HTLV-1 patients (in red) and 29 previously published sequences. The Genbank accession numbers of the new sequences from the coinfected HIV-1/HTLV-1 patients are OL546372- OL546385. The phylogeny was derived by the Neighbor-Joining method with the GTR model. Horizontal branch lengths are drawn to scale, with the bar indicating 0.005 nucleotide replacements per site. (TIF) [file pone.0271320.s001.tif]

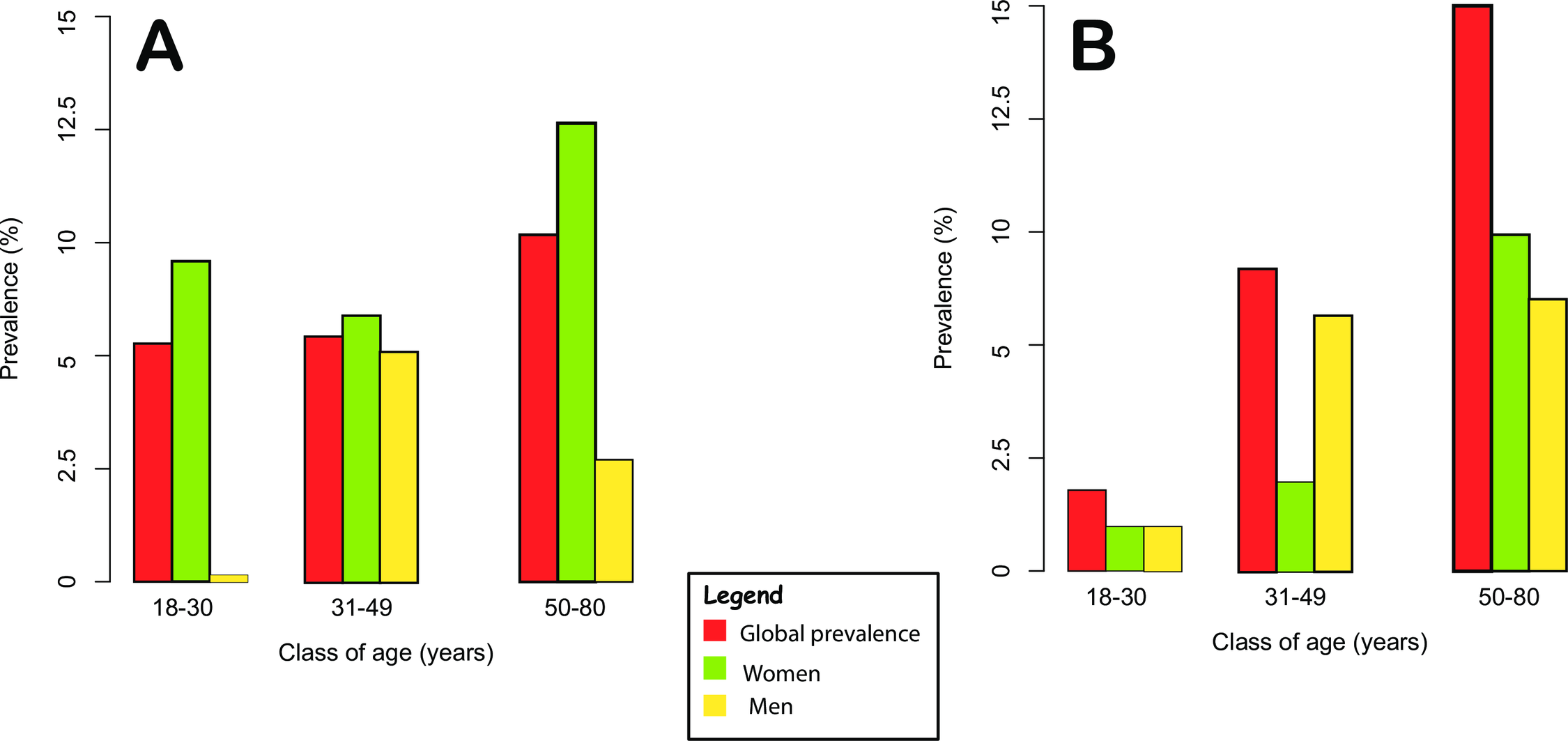

Supplement: S2 Fig — The figure shows the overall prevalence of infection by age group and the prevalence of infection by sex: A) In the HIV-1/HTLV-1 coinfected population; B) In the general population at the same place. Red color indicates global prevalence, yellow and green ones represent prevalence in men and women, respectively. (TIF) [file pone.0271320.s002.tif]
