# Supplementary material for: Human T-Lymphotropic virus type 1 and human immunodeficiency virus co-infection in rural Gabon
Source: PLoS One. 2022 Jul 22;17(7):e0271320. doi: 10.1371/journal.pone.0271320 (PMC9307203; doi:10.1371/journal.pone.0271320)
Supplement: S1 Table — (DOCX) [file pone.0271320.s003.docx]

S1 Table : Summary of the studies related to HIV-1 and HTLV-1 survey in Central Africa

| **Study** | **Country** | **Objectives** | **Population characteristics** | **Test used** | **Coinfection** | **Observations** |
| --- | --- | --- | --- | --- | --- | --- |
| (Getchell et al., 1987) | Zaïre | To isolate and identify both HTLV-I and HIV from peripheral blood lymphocytes | Total n=1 male: Age: 45 | Western blot | HTLV-1 and HIV-1, n = 2 | The patient was a 45-year-old man from Zaire who had a T4/T8 ratio of .44. |
| (Schrijvers et al., 1991) | Gabon | To study the seroprevalence of retroviral infection (HIV-1, HTLV-1) in women with different fertility statuses in Gabon | Fertile and infertile women. | Western blot | HTLV-1 and HIV-1, n = 2 | Fertility statuses : primary infertile women, secondary infertile women and fertile women |
| (Geffray et al., 1994) | Zaïre | To describe clinical and therapeutic aspects, in the relation between african histoplasmosis and AIDS | Total n=4 patients | NA | HTLV-1 and HIV-1, n = 1 | Review |
| (Mauclere et al., 1995) | Cameroon | To conduct a serological survey of HIV-1, HTLV-1 and HTLV-2 among sex workers | Total n=322  Sex workers | ELISA, IFA, WB, cocultivation with BJAB cells | HTLV-2 and HIV-1, n = 1 | Sexually transmitted disease survey |
| (Pegha Moukandja et al., 2017) | Gabon | To evaluate seroprevalence of HIV, HTVL-1, syphilis and T. gondii and rubella infection during antenatal care among women living in Franceville, Gabon. | Total n=973  Pregnant women  Mean âgé : 25.84 ± 6.9 years | ELISA, WB | HTLV-1 and HIV-1, n = 2 | Retrospective study, conducted on data collected during a cross-sectional study of HTLV-1 |
|  |  |  |  |  |  |  |

GEFFRAY, L., VEYSSIER, P., CEVALLOS, R., BEAUD, B., MAYOLLE, J., NOGIER, C., RAY, E. & THOUVENOT, D. 1994. [African histoplasmosis: clinical and therapeutic aspects, relation to AIDS. Apropos of 4 cases, including a case with HIV-1-HTLV-1 co-infection]. *Ann Med Interne (Paris),* 145**,** 424-8.

GETCHELL, J. P., HEATH, J. L., HICKS, D. R., SPORBORG, C., MANN, J. M. & MCCORMICK, J. B. 1987. Detection of human T cell leukemia virus type I and human immunodeficiency virus in cultured lymphocytes of a Zairian man with AIDS. *J Infect Dis,* 155**,** 612-6.

MAUCLERE, P., MAHIEUX, R., GARCIA-CALLEJA, J. M., SALLA, R., TEKAÏA, F., MILLAN, J., DE THÉ, G. & GESSAIN, A. 1995. A new HTLV type II subtype A isolate in an HIV type 1-infected prostitute from Cameroon, Central Africa. *AIDS Res Hum Retroviruses,* 11**,** 989-93.

PEGHA MOUKANDJA, I., NGOUNGOU, E. B., LEMAMY, G. J., BISVIGOU, U., GESSAIN, A., TOURE NDOUO, F. S., KAZANJI, M. & LEKANA-DOUKI, J. B. 2017. Non-malarial infectious diseases of antenatal care in pregnant women in Franceville, Gabon. *BMC Pregnancy Childbirth,* 17**,** 185.

SCHRIJVERS, D., DELAPORTE, E., PEETERS, M., DUPONT, A. & MEHEUS, A. 1991. Seroprevalence of retroviral infection in women with different fertility statuses in Gabon, western equatorial Africa. *J Acquir Immune Defic Syndr (1988),* 4**,** 468-70.
