# Supplementary material for: Human T-Lymphotropic virus type 1 and human immunodeficiency virus co-infection in rural Gabon
Source: PLoS One. 2022 Jul 22;17(7):e0271320. doi: 10.1371/journal.pone.0271320 (PMC9307203; doi:10.1371/journal.pone.0271320)
Supplement: Attachment — Submitted filename: Response to Reviewers.docx [file pone.0271320.s004.docx]

**Reviewer 1**-

Reviewers' comments:

Reviewer's Responses to Questions

Comments to the Author

1. Is the manuscript technically sound, and do the data support the conclusions?

The manuscript must describe a technically sound piece of scientific research with data that supports the conclusions. Experiments must have been conducted rigorously, with appropriate controls, replication, and sample sizes. The conclusions must be drawn appropriately based on the data presented.

Reviewer #1: Yes

Reviewer #2: Partly

________________________________________

2. Has the statistical analysis been performed appropriately and rigorously?

Reviewer #1: Yes

Reviewer #2: I Don't Know

_____________________________________

3. Have the authors made all data underlying the findings in their manuscript fully available?

The PLOS Data policy requires authors to make all data underlying the findings described in their manuscript fully available without restriction, with rare exception (please refer to the Data Availability Statement in the manuscript PDF file). The data should be provided as part of the manuscript or its supporting information, or deposited to a public repository. For example, in addition to summary statistics, the data points behind means, medians and variance measures should be available. If there are restrictions on publicly sharing data—e.g. participant privacy or use of data from a third party—those must be specified.

Reviewer #1: Yes

Reviewer #2: No

________________________________________

4. Is the manuscript presented in an intelligible fashion and written in standard English?

PLOS ONE does not copyedit accepted manuscripts, so the language in submitted articles must be clear, correct, and unambiguous. Any typographical or grammatical errors should be corrected at revision, so please note any specific errors here.

Reviewer #1: Yes

Reviewer #2: No

**Answer to Reviewer's Questions**

**Reviewer #1**:

In this article by Mouinga-Ondemé et al, the prevalence of HTLV-1 infection was estimated at 7.7% in an HIV-infected population in south-eastern Gabon. Dually infected HIV + HTLV patients have so far been considered at risk of a faster clinical course to AIDS, but data are badly needed to support this hypothesis. Many studies have already been published but Gabon remains one of the right place to carry out such studies due to the superposition of both endemic.

This is of major interest to better understand the factors underlying the progression of HIV in patients infected with HLTV. Unfortunately, the cross sectional aspect of the data collect considerably limits the interest of this paper. The authors proposed that plasma viral load may be slightly lower and CD4 cell count higher in double-infected patients, but these data were not statistically significant.

In addition, before to be considered for publication, various questions remain:

1 / Inclusion criteria for the population infected with HIV in this study. Explain the unbalanced sex ratio. Over representation of the pregnant women ?

**In our study, all patients enrolled were HIV-1 infected and receiving antiretroviral therapy (ART) at the only Ambulatory Treatment Centre (ATC) in Haut-Ogooué Province (Franceville is the main city). In other words, our inclusion criterion was receiving treatment at the Centre. We found that the majority of patients followed at the ATC in Franceville were women (209 women and 90 men). This observation is coherent with the study conducted by Liégeois and colleagues in 2012 (**[**http://dx.doi.org/10.7448/IAS.15.2.17985**](http://dx.doi.org/10.7448/IAS.15.2.17985)**) who showed that the majority of patients followed in the ATC are women. We think this situation may be explained by the fact that men prefer to go to other cities for treatment for fear of being stigmatized. No pregnant women were enrolled in the study. In the discussion section, we raises the concern of vertical transmission of HTLV-1 via breastfeeding in HIV-1/HTLV-1 women, as WHO developed programs to prevent the vertical transmission of HIV from mother to child, especially by using antiretroviral drugs (WHO, 2016).**

2 / EIA + but PCR negative and / or WB not known: additional molecular tests are absolutely necessary in this country with a high STLV / HTLV prevalence. Have you performed additional seological and molecular tests?

**We thank the reviewer for this question. We used the screening strategy commonly used to detect HTLV-1 and 2 and published in many reports (e.g., Djuicy et al., 2018** [**https://doi.org/10.1371/journal.pntd.0006832**](https://doi.org/10.1371/journal.pntd.0006832)**; Ramassamy et al., 2020 doi:10.1111/trf.15838). We acknowledge that other options include additional assays such as the INNO-LIA HTLV I/II Score (Immunogenetics, Gent, Belgium), but we did not do this because we used western blot. In addition, as we discussed in this paper, options are limited for HTLV diagnostic in resource-limited countries. The EIA test used we for serology (HTLV-I/II ELISA 4.0, MP Biomedicals) has high specificity. Because of false EIA+ specimen, we used the WB test for serological confirmation or to discriminate between HTLV-1 and HTLV-2. Without WB results, we consider that PCR results for conserved regions of the genome (tax) and sufficiently different regions of the genome (Env) confirmed or refuted the results of the EIA.**

3 / The assays used for the RT-PCR viral load and the CD4 count must be specified (manufacturers?)

**As recommended by the reviewer, we added sentences which describe the assays used for the CD4 count and the RT-PCR viral load. Please see Methods section, line 106 to 109 in the new version of manuscript.**

4 / Could we have more details about HTLV sequencing? Is the population only infected with HTLV-1 B?

**As requested by the reviewers, we provided HTLV sequencing data in the supplementary figure. First, we successfully amplified the PTLV tax region for 23 specimens; however, only 14 specimens had sufficient DNA to amplify a fragment of the Env gene (for more information see the line 135 to 142 of the new version of the manuscript). The phylogenetic tree, in supplementary data, shows that most of the study population is infected with HTLV-1B. Only one patient is infected with HTLV-1D (****Gab-2276FCV).**

5 / Clinical data concerning bi or mono infected patients are essential. HAM / TSP / ATTL reported in dually infected patients? Or more frequent neurological or haematological pathologies in this group?

**We agree with the reviewer that clinical data concerning the study population are essential. None of the participants included in this study presented signs of TSP / HAM and/or ATLL. Unfortunately, we did not have clinical and epidemiological data for both mono and bi infected groups. Only some bacteriological, virological and parasitological opportunistic diseases have been reported in mono and coinfected patients. There were all correlated with the rate of CD4. We did not find it appropriate to discuss this because the purpose of our study was investigated HTLV-1 and HIV-1 co-infection in Haut-Ogooué Province. We agree that this merits further investigations in the future.**

The study is currently limited by the cross-sectional approach and the scarcity of biological and clinical data.

Suggestion to Authors: Despite the nucleotide diversity between HIV-1 and HTLV-1, their RT enzymes exhibit a fairly similar structure in the palm and fingers. All of these patients are (or have been) treated with 3TC / TFC and 184V and must certainly be present in HIV-RT in the majority of patients. A short sequencing of the HTLV RT pocket could be interesting because the intracellular level of lamivudine will lead to a selection of certain HTLV mutations; V118I and others have been proposed as relevant in explaining the high "resistance" of HTLV to 3TC in the 1980s. But recent interest in resistance to HTLV 3TC at least in vitro (DOI: 10.1086 / 322785) has shown that the question is still open. As the buffy coat was collected, sequencing could be easly performed. The presence of RT 3TC / FTC mutation (s) will confirm a potential replication of HTLV at the cellular level in this doubly infected population and will add original data to this article.

**The suggestion made by the reviewer is very relevant. Unfortunately, plasma and buffy coat collected from these patients, in 2018 and 2019, was also used for several other studies. After sequencing the HTLV Env gene, no more DNA was available from several patients for further investigations. Finally, we were unable to amplify and sequence HTLV RT. We note this approach for our future studies.**

**In contrast, we amplified HIV RT to check some mutations in HIV-1/HTLV-1 coinfected patients. The majority of the** **HIV-1/HTLV-1 co-infected patients had a viral load suppression (VL<2.5 Log); Only one patient had a virological failure (the one co-infected with HTLV-1D). The HIV-1 RT region of 646 bp infected this patient was successfully sequenced. This patient was receiving ABC+3TC+EFV first-line antiretroviral therapy. Several HIV drug resistance mutations (DRMs) were identified: L74I, V75T, Y115F, M184V (NRTI), K103N, Y181C (NNRTI). Please see line 209 to 214.**

**Reviewer #2: 480 / 5000**

The article does not have sufficient consistency to support publication in Plos One.

Although the confirmation of HIV/HTLV-1 co-infection in patients from Gabon (Central Africa) is a serious public health problem, from a technical-scientific point of view the objectives are not clearly described, as well as the methodology and, furthermore, the results are insufficient for a publication in Plos One.

Therefore, I recommend that the article be submitted to another journal.

________________________________________
