# Supplementary material for: Human T-Lymphotropic virus type 1 and human immunodeficiency virus co-infection in rural Gabon
Source: PLoS One. 2022 Jul 22;17(7):e0271320. doi: 10.1371/journal.pone.0271320 (PMC9307203; doi:10.1371/journal.pone.0271320)
Supplement: Attachment — Submitted filename: Response to Reviewers.docx [file pone.0271320.s005.docx]

**Dr. MOUINGA-ONDÉMÉ Augustin**

Unité des Infections Rétrovirales et Pathologies Associées,

Centre International de Recherches Médicales de Franceville (CIRMF),

B.P. 769, Franceville, Gabon. [ondeme@yahoo.fr](mailto:ondeme@yahoo.fr)

Dear Editor,

Please find attached a revised version of the manuscript (PONE-D-21-19756R1) entitled **“Human T-Lymphotropic virus type 1 and Human Immunodeficiency Virus co-infection in rural Gabon”** by Mouinga-Ondémé and colleagues. We thank you very much for facilitating this set of revisions to our paper.

We appreciate the reviewers’s diligence and we thank them for their constructive and helpful comments on the manuscript. We have modified our manuscript accordingly as detailed below.

Corrections in the new manuscript are highlighted in yellow. Concerning the illustrations, we have added supplementary data: the figure (S2 Fig) and the table (S1 table) as suggested by the reviewers.

We have addressed all the points raised by Reviewers #1 and #2 made the required corrections to the manuscript.

Given the new data analysis, we added a new author with approval from all. Delia Doreen DJUICY is now a co-author of this paper, she was added at 7^th^ place.

We hope that these modifications to the manuscript sufficiently addresses the concerns mentioned by the reviewers and we thank them for their valuable advices.

Yours Sincerely,

Dr. Augustin Mouinga Ondémé

**Letter**

PONE-D-21-19756R1

Human T-Lymphotropic virus type 1 and Human Immunodeficiency Virus co-infection in rural Gabon

PLOS ONE

Dear Dr. Mouinga-Ondemé,

Thank you for submitting your manuscript to PLOS ONE. After careful consideration, we feel that it has merit but does not fully meet PLOS ONE’s publication criteria as it currently stands. Therefore, we invite you to submit a revised version of the manuscript that addresses the points raised during the review process.

Please submit your revised manuscript by May 06 2022 11:59PM. If you will need more time than this to complete your revisions, please reply to this message or contact the journal office at [plosone@plos.org](mailto:plosone@plos.org). When you're ready to submit your revision, log on to https://www.editorialmanager.com/pone/ and select the 'Submissions Needing Revision' folder to locate your manuscript file.

Please include the following items when submitting your revised manuscript:

- A rebuttal letter that responds to each point raised by the academic editor and reviewer(s). You should upload this letter as a separate file labeled 'Response to Reviewers'.
- A marked-up copy of your manuscript that highlights changes made to the original version. You should upload this as a separate file labeled 'Revised Manuscript with Track Changes'.
- An unmarked version of your revised paper without tracked changes. You should upload this as a separate file labeled 'Manuscript'.

If you would like to make changes to your financial disclosure, please include your updated statement in your cover letter. Guidelines for resubmitting your figure files are available below the reviewer comments at the end of this letter.

If applicable, we recommend that you deposit your laboratory protocols in protocols.io to enhance the reproducibility of your results. Protocols.io assigns your protocol its own identifier (DOI) so that it can be cited independently in the future. For instructions see: <https://journals.plos.org/plosone/s/submission-guidelines#loc-laboratory-protocols>. Additionally, PLOS ONE offers an option for publishing peer-reviewed Lab Protocol articles, which describe protocols hosted on protocols.io. Read more information on sharing protocols at <https://plos.org/protocols?utm_medium=editorial-email&utm_source=authorletters&utm_campaign=protocols>.

We look forward to receiving your revised manuscript.

Kind regards,

Fatah Kashanchi
Academic Editor
PLOS ONE

Journal Requirements:

Please review your reference list to ensure that it is complete and correct. If you have cited papers that have been retracted, please include the rationale for doing so in the manuscript text, or remove these references and replace them with relevant current references. Any changes to the reference list should be mentioned in the rebuttal letter that accompanies your revised manuscript. If you need to cite a retracted article, indicate the article’s retracted status in the References list and also include a citation and full reference for the retraction notice.

Additional Editor Comments:

Please address the Reviewers comments


[Note: HTML markup is below. Please do not edit.]

Reviewers' comments:

Reviewer's Responses to Questions

**Comments to the Author**

1. If the authors have adequately addressed your comments raised in a previous round of review and you feel that this manuscript is now acceptable for publication, you may indicate that here to bypass the “Comments to the Author” section, enter your conflict of interest statement in the “Confidential to Editor” section, and submit your "Accept" recommendation.

Reviewer #1: (No Response)

Reviewer #2: All comments have been addressed

2. Is the manuscript technically sound, and do the data support the conclusions?

The manuscript must describe a technically sound piece of scientific research with data that supports the conclusions. Experiments must have been conducted rigorously, with appropriate controls, replication, and sample sizes. The conclusions must be drawn appropriately based on the data presented.

Reviewer #1: (No Response)

Reviewer #2: Partly

3. Has the statistical analysis been performed appropriately and rigorously?

Reviewer #1: (No Response)

Reviewer #2: Yes

4. Have the authors made all data underlying the findings in their manuscript fully available?

The [PLOS Data policy](http://www.plosone.org/static/policies.action#sharing) requires authors to make all data underlying the findings described in their manuscript fully available without restriction, with rare exception (please refer to the Data Availability Statement in the manuscript PDF file). The data should be provided as part of the manuscript or its supporting information, or deposited to a public repository. For example, in addition to summary statistics, the data points behind means, medians and variance measures should be available. If there are restrictions on publicly sharing data—e.g. participant privacy or use of data from a third party—those must be specified.

Reviewer #1: (No Response)

Reviewer #2: Yes

5. Is the manuscript presented in an intelligible fashion and written in standard English?

PLOS ONE does not copyedit accepted manuscripts, so the language in submitted articles must be clear, correct, and unambiguous. Any typographical or grammatical errors should be corrected at revision, so please note any specific errors here.

Reviewer #1: (No Response)

Reviewer #2: Yes

6. Review Comments to the Author

Please use the space provided to explain your answers to the questions above. You may also include additional comments for the author, including concerns about dual publication, research ethics, or publication ethics. (Please upload your review as an attachment if it exceeds 20,000 characters)

**Answer to Reviewer's Questions**

**Reviewer #1**

Manuscript PONE-D-21-19756R1, Co-infection with human lymphotropic T virus type 1 and human immunodeficiency virus in rural Gabon is a descriptive cross-sectional study. The lack of clarity regarding the objectives and the lack of basic clinical data without any biological and clinical problems are major limitations to its publication in PlosOne
Despite these major limitations, given the importance of HIV / HTLV co-infection in public health and research and the paucity of studies, it is important to report these epidemiological data to the general public of PlosOne.

We suggest:

Improve the quality of the prevalence of coinfection data by matching the HIV HTLV population with the general population of the same place in the country in order to compare the prevalence of these two retroviruses in these different populations. This could at least lead to showing that the prevalence of HTLV, as expected, is higher in the HIV-infected population.

**We agree with the Reviewer for improving the quality of the prevalence. Concerning general population of the same place of the study, prevalence was obtained from data used for our previous study published by Djuicy et *al*. (doi.org/10.1371/journal.pntd.0006832). A paragraph was added in the discussion section from the lines 281 to 285. Also, a supporting information file, S2 Fig. B, about the HTLV-1 prevalence in the general population, was provided and mentionned at the line 284.**

And to make this article more attractive, we suggest that the discussion include a review of the literature on this double infection. A summary in the form of a table of epidemiological studies on HIV / HTLV co-infections which comment on the specific situation in Central Africa would be very necessary.

**We thank the Reviewer for this suggestion. At the line 299 to 300, we mentionned that « Although the prevalence of these two retroviruses is very high in many African countries, limited number of studies have been carried on the co-infection.” on the specific situation in Central Africa, we do not find published studies about HIV-1/HTLV-1 co-infection. We found only studies based on serological survey or seroprevalence evaluation of HTLV-1, HIV-1 and others pathogens in different populations; and some of these studies are summarized in the S1 table. Lines 307 to 311.**

**Reviewer #2**

In this new version the manuscript presents sufficient consistency to support publication in the Plos One. However some modifications are necessary.
The title of the article sounds appropriate. However the data presented do not support the hypothesis the factors evaluated may favor the rapid evolution and progression to AIDS in HTLV-1/ HIV co-infected patients. This hypothesis must be reviewed since the corresponding author informed to reviewer #1 it is not possible to include new evaluations using the plasma and buffy coat.

Points to improve:

- Line 100: Please define ART.

**As recommended by the Reviewer, ART was defined as « antiretroviral therapy » (line 101 in the new manuscript version).**

- Line 114: Western Blot: please write Western with initial capital letter.

**As recommended by the Reviewer, « Western » was written with initial capital letter (line 132).**

- Line 130: Please define ANRS.

**We agree with the Reviewer, and ANRS was defined as « French National Agency for Research on AIDS and Viral Hepatitis. » (lines 122 to 123).**

METHODS

The methodology should be described more organized and coherent. A good way is to insert subtitles:
1.Study design an population
2.HIV Viral Load and CD4 counts
3.HTLV diagnosis (include serological and molecular diagnosis. e.g.: serological diagnosis was performed..../ Molecular diagnosis was performed for all samples with a positive ELISA result. We extracted viral DNA from buffy-coat. )
4.HIV diagnosis

**We agree with the Reviewer. The METHODS section was edited and organized as recommended :**

**1. Study design and population (Line 96)**

**2. HIV Viral Load and CD4 counts (Line 107)**

**3. HIV diagnosis (Line 116)**

**4. HTLV diagnosis (Line 127)**

**5. Phylogenetic analysis (Line154)**

**6. Statistical analysis (Line 160)**

- Line 150: The study was approved ...(It would be better moving this topic to the end of subtitle 1.: “Study design an population”

**As recommended by the Reviewer, this topic was moved to the end of « Study design and population » section (lines 104 to 105)**

RESULTS

- Patient Population: Please, describe the results in the order they appear in the table 1. This will make it easier for the reader to follow the data.

**We agree with the Reviewer. Results was organized by following the order they appear in the table 1 (lines 173 to 175)**

I suggest adding a bar graph figure of HTLV-1 prevalence PCR among men, women and total, according to the age groups evaluated.

**As suggested by the Reviewer, a bar graph has been added as a supplementary data (S2 Fig. A), and this reference was inserted in the main text (line 218).**

DISCUSSION
It is important to review some points of the discussion after reviewing of the hypothesis that factors evaluated may favor the rapid evolution and progression to AIDS in HTLV-1/ HIV co-infected patients.

**We agree with the Reviewer. In the discussion section, a paragraph was added at the lines 270 to 277**

7. PLOS authors have the option to publish the peer review history of their article ([what does this mean?](https://journals.plos.org/plosone/s/editorial-and-peer-review-process#loc-peer-review-history)). If published, this will include your full peer review and any attached files.


If you choose “no”, your identity will remain anonymous but your review may still be made public.


**Do you want your identity to be public for this peer review?** For information about this choice, including consent withdrawal, please see our [Privacy Policy](https://www.plos.org/privacy-policy).

Reviewer #1: No

Reviewer #2: No
